# Are progressive shoulder exercises feasible in patients with glenohumeral osteoarthritis or rotator cuff tear arthropathy?

**DOI:** 10.1186/s40814-022-01127-8

**Published:** 2022-08-03

**Authors:** Josefine Beck Larsen, Helle Kvistgaard Østergaard, Theis Muncholm Thillemann, Thomas Falstie-Jensen, Lisa Cecilie Urup Reimer, Sidsel Noe, Steen Lund Jensen, Inger Mechlenburg

**Affiliations:** 1 Department of Orthopaedic Surgery, Aarhus University Hospital, Aarhus N, Denmark; 2 Department of Clinical Medicine, Aarhus University, Aarhus N, Denmark; 3 Department of Orthopeadic Surgery, Viborg Regional Hospital, Viborg, Denmark; 4 Interdisciplinary Orthopaedics, Aalborg University Hospital, Aalborg, Denmark; 5 Department of Clinical Medicine, Aalborg University, Aalborg, Denmark

**Keywords:** Glenohumeral osteoarthritis, Rotator cuff tear arthropathy, Shoulder, Exercise, Total shoulder replacement

## Abstract

**Background:**

Little is known about the feasibility of progressive shoulder exercises (PSE) for patients with glenohumeral osteoarthritis (OA) or rotator cuff tear arthropathy (CTA). The aim of this study was to investigate whether 12 weeks of PSE is feasible in patients with glenohumeral OA or CTA eligible for shoulder arthroplasty. Moreover, to report changes in shoulder function and range of motion (ROM) following the exercise program.

**Methods:**

Twenty patients were included. Eighteen patients (11 women, 15 with OA), mean age 70 years (range 57–80), performed 12 weeks of PSE with one weekly physiotherapist-supervised and two weekly home-based sessions. Feasibility was measured by dropout rate, adverse events, pain, and adherence to PSE. At baseline and end of intervention, patients completed the Western Ontario Osteoarthritis of the Shoulder (WOOS) score and Disabilities of the Arm, Shoulder and Hand (DASH). Data to assess feasibility were analyzed using descriptive statistics.

**Results:**

Two patients dropped out and no adverse events were observed. Sixteen of the eighteen patients (89%) had a high adherence (≥ 70%) to the physiotherapist-supervised sessions. Acceptable pain levels were reported; in 76% of all exercise sessions with no numeric rating scale (NRS) score over five for any exercise. WOOS improved with a mean of 23 points (95% CI 13;33), and DASH improved with a mean of 13 points (95% CI 6;19).

**Conclusion:**

Adherence to PSE was high and dropout rates were low. PSE is feasible, safe and may relieve shoulder pain, improve function and ROM in patients with glenohumeral OA or CTA. The patient-experienced gains after PSE seem clinically relevant and should be compared to arthroplasty surgery in a RCT setting.

**Trial registration:**

According to Danish law, this study did not need an approval by the Central Denmark Region Committee on Health Research Ethics. Approval from The Danish Data Protection Agency (journal number 1-16-02-15-20) was obtained.

## Key messages regarding feasibility

- Uncertainties: the present study warrants further investigation in a randomized controlled trial design, as the results suggest that patients can achieve clinically meaningful improvements in patient-reported pain, shoulder function and objectively measured range of motion.
- Key feasibility findings: it is feasible to perform progressive shoulder exercises in patients with glenohumeral OA and CTA eligible for total shoulder replacement.
- Implications: progressive shoulder exercises with emphasis on not increasing pain after completing exercises seem to be a potentially effective treatment for patients with glenohumeral OA and CTA.

## Background

Glenohumeral osteoarthritis (OA) and rotator cuff tear arthropathy (CTA) are well-known causes of pain and weakness in the shoulder joint reducing activities of daily living and quality of life [1–5]. Treatment of glenohumeral OA and CTA is primarily aimed at reducing pain and secondly at restoring function. First line treatment is non-surgical including activity modification, exercise or physiotherapy; second line treatment is pharmacological including oral pain medication and corticosteroid injections [1, 6]. If non-surgical treatment fails, the third and final line of treatment is total anatomical shoulder arthroplasty (TSA) or reverse shoulder arthroplasty (RSA), respectively [7].

Currently, evidence of the effectiveness of exercise interventions in management of glenohumeral OA and CTA is limited [8]. Exercise interventions have been shown to be effective in patients with massive rotator cuff tears (MRCT), however, patients with degenerative changes in the glenohumeral joint are excluded from these studies [9–14]. A recent systematic review and meta-analysis found that exercise is as effective as surgery (cuff-repair) for improving quality of life, disability, and pain in patients with MRCT, however with low certainty of the evidence [14]. They also found that the content of reporting exercise interventions in the studies was extremely poor. The exercise intervention in the studies varied from individually tailored exercise programs to hand cyclo-ergometer, physiotherapy, and home-based exercises. Often exercises focused on shoulder muscle performance exercises, stretching, posture, and scapular correction [14]. Studies of MRCT exercise often focus on flexion, i.e., the reeducation of the deltoid muscle and on the external rotation [12]. Several studies and reviews give suggestions for physiotherapy for patients with glenohumeral OA, however, these suggestions are based on research performed on shoulder impingement and knee OA. They all suggest that physiotherapy should be tailored to the individual but overall consist of improvement of range of motion (ROM) and strengthening of the rotator cuff and scapula-thoracic muscles [2, 15, 16]. Similarly, exercise has been shown to potentially postpone or even eliminate the need for arthroplasty in patients with knee OA, exercise has shown to have a significant impact on patient symptoms, physical function, intake of analgesics and sick leave [17]. Moreover, the effectiveness of exercise therapy is currently under investigation in hip OA [18].

Several studies including a Cochrane review have suggested the need for trials comparing total shoulder arthroplasty to non-surgical treatment [1, 7, 8, 19]. Thus, feasibility and safety of an exercise intervention must be established prior to conducting randomized controlled trials.

The primary aim of this study was to examine if a 12-week program of progressive shoulder exercises is feasible concerning adherence to exercise, pain response, adverse events, and dropout rate in patients with primary glenohumeral OA or CTA. The secondary aim was to report changes in patient-reported shoulder function and range of motion (ROM) after completion of the exercise program.

## Methods

### Study design

The feasibility study was conducted at the orthopedic departments at Aarhus University Hospital, Aalborg University Hospital and Viborg Regional Hospital in Denmark. The study was conducted in accordance with the Declaration of Helsinki. The study protocol was forwarded to the Central Denmark Region Committee on Health Research Ethics. According to Danish law, this type of study does not need an ethics approval (journal number 1-10-72-286-19). Approval from The Danish Data Protection Agency (journal number 1-16-02-15-20) was obtained. All patients gave written informed consent prior to inclusion.

### Inclusion and exclusion criteria

Patients ≥ 55 years with moderate to severe glenohumeral OA eligible for TSA or patients ≥ 65 years with CTA (Hamada > 2) eligible for RSA were invited. Grade of glenohumeral OA was assessed by the Samilson and Prietro classification by measuring the lower osteophyte on a standard anterior-posterior radiograph [20]. Degree of CTA was classified according to the Hamada classification [1–5] based on a standard anterior-posterior radiograph [21]. Patients were not included if complying with the following exclusion criteria: (1) previous proximal humerus fracture; (2) planned other upper extremity surgery within six months; (3) rheumatoid arthritis and other types of arthritis not diagnosed as primary glenohumeral OA; (4) cancer diagnosis and receiving chemotherapy, immunotherapy or radiotherapy, (5) other reasons (i.e., cognitive deficits preventing participation).

### Intervention

The patients followed a standardized exercise intervention developed and described in accordance with Consensus on Exercise Reporting Template (CERT) [22]. The exercise intervention consisted of a 12-week progressive shoulder exercises program with three weekly exercise sessions: one physiotherapist-supervised session and two home-based sessions [23]. Patients in groups of one to four were scheduled for 12 physiotherapist-supervised sessions of approximately 45–60 min per session over a 12-week period at the three participating hospitals. The supervising physiotherapists were introduced to the intervention and the written material containing information on how to practically introduce the individual exercises including how to progress, regress and correct exercises. The intervention was delivered in accordance with the training protocol. If a patient reported excessive pain, individual adaptations and regress in exercises and load were made. Adaptations were registered in the physiotherapists’ training log. The physiotherapists used verbal encouragement and motivation and manually guided the patient when performing the exercises if needed.

The development of the progressive shoulder exercises program was based on current knowledge of characteristic physical impairments in individuals with glenohumeral OA and CTA. It consisted of exercises targeting shoulder range of motion (ROM) and muscle strength. The exercise program is described in Table 1 and included pendulum exercises and table slides as warm up and strengthening exercises in external rotation, internal rotation, posture exercise, abduction, and forward flexion. The exercise program hand out to the patients with pictures of the exercises are available in the supplementary material in the protocol describing the randomized controlled trial [23]. The strengthening exercises were performed with as much range as possible in sets of three with 8–12 repetitions followed by 60 s of rest. The intensity increased progressively from 12 repetitions and three sets the first 2 weeks to 8 repetitions and three sets in the last 2 weeks.

**Table 1:** Description of the exercises used in the exercise program

Exercise ***Muscle(s) targeted***	Performance
**Pendulum** (warm up)	Good hand on the back of a chair and a little bent over. Make 10 small circles clockwise and counterclockwise.
**Table slides** (warm up)	Patient sitting at a table with the hand on a dry cloth. Make 10 forward slides, make 10 large circles and 10 figure eights.
**External rotation** *Infraspinatus* *Teres minor* *Deltoideus posterior*	Level B: Patient sitting with elbow at 90°. Make external rotation. Level A: Patient standing with elbow at 90°. Make external rotation.
**Internal rotation** *Subscapularis* *Teres major*	Level B: Patient sitting with elbow at 90 . Make internal rotation. Level A: Patient standing with elbow at 90 . Make internal rotation.
**Posture exercise** *Trapezius*	Level B: Patients standing with arms hanging along the body. Squeeze the shoulder blades together. Level A: Patient standing with arms in 45° forward flexion. Make extension with elbows stretched while squeezing the shoulder blades together.
**Abduction** *Supraspinatus* *Deltoid*	Level B: Patient standing facing a wall. Make a slight forward flexion and eccentric abduction. Level A: Patient standing with arm along the sides. Make abduction.
**Forward flexion** *Deltoid*	Level B: Patient supine with arms along the sides. Make forward flexion. Level A: Patient standing with arms in 45° forward flexion. Make forward flexion.

Patients were advised to conduct the progressive shoulder exercises program three times a week and no more than four times per week to ensure recovery between training sessions. If patients cancelled the supervised exercise sessions, they were advised to conduct the session at home. The planned progression depended on pain level during exercise rated on a 0–10-point numeric rating scale (NRS) after each exercise. Pain was considered unacceptable if pain exceeded five on the NRS; in this case, level or load of exercises was regressed [24]. However, some patients reported NRS pain of five at rest and it was thus impossible to perform the exercises with a NRS below five. Therefore, patients reporting high initial NRS were advised that increase of shoulder pain during exercise was acceptable if the pain quickly decreased to resting level after finishing the exercise. The equipment used for the intervention were TheraBand® elastic bands (Akron, USA). Following completion of the 12-week progressive shoulder exercise program, patients were encouraged to continue the exercises.

### Outcome measures

Outcome assessment was performed at baseline and 12 weeks after completion of the progressive shoulder exercise program. All baseline and follow-up assessments were performed by one of three project physiotherapists at the three hospitals.

### Primary outcomes

The primary outcomes to assess feasibility were adherence to the exercise program, pain response to the exercise intervention, adverse events and dropout rate.

### Adherence

Adherence to the exercise program was registered by the supervising physiotherapists while adherence to the home-based exercises was registered by the patients in an exercise log. High adherence to the exercise program was a priori defined as participation in > 70% (8 out of 12 sessions) of the supervised exercise sessions; this was similar to other exercise intervention studies [18, 24, 25].

### Pain response

Patients rated their pain levels on a 10-point NRS at rest in the beginning of each supervised exercise session and between second and third exercise set. In their exercise log, patients further reported the average pain level during exercises conducted at home and pain level two hours after completing the exercise sessions.

### Adverse events

Adverse events or harms were defined as any unintended and unfavorable sign, symptom or disease occurring during the period from inclusion until 12-week follow-up resulting in contact with the healthcare system regardless of a causal relationship with the intervention and event. Serious adverse events were categorized in accordance with the definitions established by the United States Food and Drug Administration: (1) death; (2) life-threatening; (3) hospitalization; (4) disability or permanent damage; (5) congenital anomaly/birth defect; (6) required intervention to prevent permanent impairment or damage; (7) other serious (important medical events) [26].

### Patient feedback

Patients were asked to give feedback in group interviews on the exercise intervention, transportation to training facilities, outcome measures and willingness to undergo randomization to either shoulder arthroplasty surgery or exercise.

### Secondary outcomes

Patient-reported outcomes were measured at baseline and at 12 weeks. The most important patient-reported outcome was the Western Ontario Osteoarthritis of the Shoulder (WOOS) score followed by Disabilities of the Arm, Shoulder and Hand (DASH). Other patient-reported outcomes included pain intensity, use of analgesics during the previous week, EQ-5D-5L [27] and subjective shoulder value (SSV).

### Patient-reported outcomes

WOOS is a valid, reliable and responsive outcome for patients with glenohumeral OA [28]. WOOS is a measurement of shoulder-related quality of life in patients with OA. It provides scores on four domains: (1) physical symptoms; (2) sport, recreation, and work; (3) lifestyle; and (4) emotions. Responses to each question are given using a visual analogue scale with a possible score ranging from 0 to 100 (0 best 100 worst).

DASH is designed to evaluate multiple disorders in the upper limp. DASH has been shown to be valid and responsive compared to other specific measures in the upper limb [29]. DASH is a self-administered questionnaire consisting of 30 core questions and eight questions assessing work, sports and/or performing arts activities. Each individual item is scored on a 5-point Likert scale, and lower scores correlate to minimal impairments. The cumulative DASH score is scaled from 0–100 with higher scores indicating a higher degree of disability [30].

The EQ-5D-5L is a health-related questionnaire measuring quality of life on five dimensions: Mobility, self-care, daily activities, pain/discomfort, and anxiety/depression. Each dimension consists of one item divided into five levels (no, slight, moderate, severe problems, unable to do) [27].

Pain was measured using a 100-mm visual analogue scale (VAS). Patients were asked to rate pain intensity at rest and during activity (VAS during the last week). Furthermore, patients were asked whether they had nightly pain during the last week (yes/no).

SSV is defined as a patient’s subjective shoulder assessment expressed as a percentage of an entirely normal shoulder, representing a score of 100%. The SSV is an easily administered, responsive and valid measure of shoulder function [31].

### Objective outcome

Active ROM was measured with a goniometer in abduction and flexion. Assessment of ROM is reliable provided the measurement instrument and the patient position is standardised [32, 33].

### Baseline data

Characteristics related to sex, age, height, weight, marital status, educational level, employment status, place of residency, substance abuse (alcohol and smoking), and duration of shoulder symptoms were registered for all patients.

### Data analysis

Adherence to the exercise intervention was calculated in percent for both the number of supervised exercise sessions and the total number of exercise sessions per week. The percentage was then presented as a mean (SD) adherence, with *N* (%) of patients with high adherence. Pain response during the exercise sessions was reported as a percentage.

$${\mathrm{Adherence}}_{\mathrm{supervised}\ \mathrm{exercise}\ \mathrm{sessions}}=\frac{\mathrm{Number}\ \mathrm{of}\ \mathrm{attended}\ \mathrm{supervised}\ \mathrm{sessions}}{12}$$

$${\mathrm{Adherence}}_{\ge 3\ \mathrm{exercise}\ \mathrm{sessions}\ \mathrm{per}\ \mathrm{week}}=\frac{\mathrm{Number}\ \mathrm{of}\ \mathrm{weeks}\ \mathrm{with}\ge 3\ \mathrm{exercise}\ \mathrm{sessions}}{12}$$

$${\mathrm{Percentage}}_{\mathrm{experiencing}\ \mathrm{NRS}>5}=\frac{\mathrm{Number}\ \mathrm{of}\ \mathrm{sessions}\ \mathrm{with}\ \mathrm{pain}\ \mathrm{score}>5}{\mathrm{Total}\ \mathrm{number}\ \mathrm{of}\ \mathrm{exercise}\ \mathrm{sessions}}$$

Changes from baseline to follow-up were analysed with a paired t-test in normally distributed data (QQ plots and histograms), and when data was not normally distributed, the Wilcoxon signed-rank test was used. The level of significance was 0.05 and the STATA 17.0 (StataCorp., College Station, TX, USA) software package was used for the data analysis.

Patient feedback from the focus group interviews was organized into categories related to expectations to the exercise intervention, transportation to training facilities, outcome measures, and willingness to undergo randomisation to shoulder arthroplasty surgery or exercise. The group interviews were conducted with seven of the 18 patients: one group with four patients and one group with three. The seven patients had completed between 9 and 12 weeks of the intervention at the time of the interview.

## Results

### Eligible patients

**Fig. 1:**
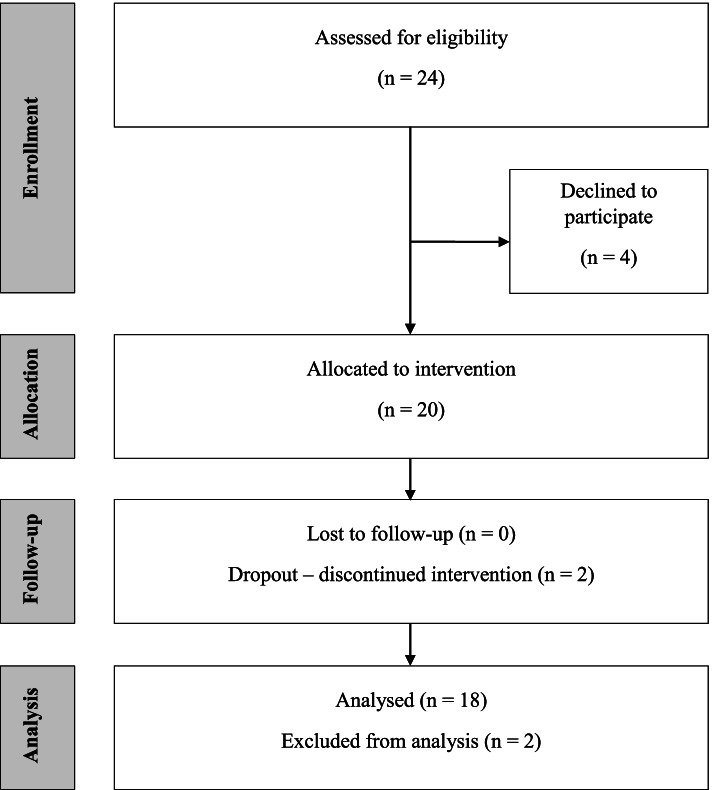
Flow diagram of patient enrollment, allocation, and follow-up analysis

Twenty-four patients were assessed for eligibility from 15th of April until 01st of September 2020, and 20 patients (15 eligible for TSA) were included (Fig. 1). Eighteen patients completed the 12 weeks of exercise. The patients were aged 69 ± 7.2 years and 11 were females.

Baseline demographic and clinical characteristics of participants are presented in Table 2. Comorbidities included asthma, atrial fibrillation, Parkinson’s disease, glaucoma, fibromyalgia, and hypertension. All patients had used pain-relief medication with paracetamol weekly or daily for their shoulder pain, four patients also nonsteroidal anti-inflammatory drugs and three patients took opioids. Three patients had previously undergone shoulder surgery (one subacromial decompression, one capsular release, one rotator cuff repair) and 12 patients had previously tried physiotherapy and/or exercise.

**Table 2:** Baseline demographics and clinical characteristics of the 20 included patients

Characteristics	
Gender, female/male	11/9
Age, years	69 ± 7.2
Body mass index, kg/m^2^	28.4 ± 3.6
Handedness, right/left	20/0
Shoulder undergoing surgery, right/left	10/10
Marital status^c^	
*Cohabiting or married*	18
*Widow/widower*	1
Comorbidities, yes/no	9/11
Alcohol intake^a^	
*Below recommended*	15
*Above recommended*	5
Smoking	
*Non-smokers*	19
*Smokers*	1
Education^b^	
*Low*	6
*Medium*	4
*High*	9
Employment status^a^	
*Employed*	5
*Outside the labor market (disability benefit)*	1
*Retired*	14
Duration of shoulder symptoms	
*6 months–2 years*	3
*2–5 years*	10
*> 5 years*	7

^a^Recommended alcohol intake for men is 14 standard units of alcohol per week and for women 7 standard units of alcohol per week

^b^Education [34] was defined as low = *none–lower secondary, medium = upper-secondary–non-tertiary, and high = tertiary education*

^c^Data were missing in one patient

### Feasibility and adherence

The results for each aspect of the primary aim of feasibility are provided in Table 3. The mean adherence to the supervised exercise sessions was 89% (SD 12%). Sixteen patients had a high adherence defined as participation in ≤ 70% of the supervised exercise sessions. The main reason for non-adherence was vacation. The mean adherence to the total number of exercise sessions per week (three or more) was 82% (SD 17%). Fourteen patients had a high adherence to the total number of weekly exercise sessions. No patients dropped out due to musculoskeletal injury.

**Table 3:** Feasibility and adherence of the 18 patients

Primary aim	Results
Adherence to the supervised exercise sessions, mean (SD) *N (%)*	High 89% (12%) 16 (89%) patients participated in > 70% (8 out of 12 sessions) of the supervised exercise sessions
Adherence to three or more exercise sessions per week, mean (SD) *N (%)*	High 82% (17%) 14 (78%) patients completed three or more weekly exercise sessions in 8 out of 12 weeks
Adverse events	No adverse events
Drop-out rate	2 patients dropped out One due to social circumstances and other illness after the second supervised exercise session. One due to additional pain after the first supervised exercise session

*SD* standard deviation

**Fig. 2:**
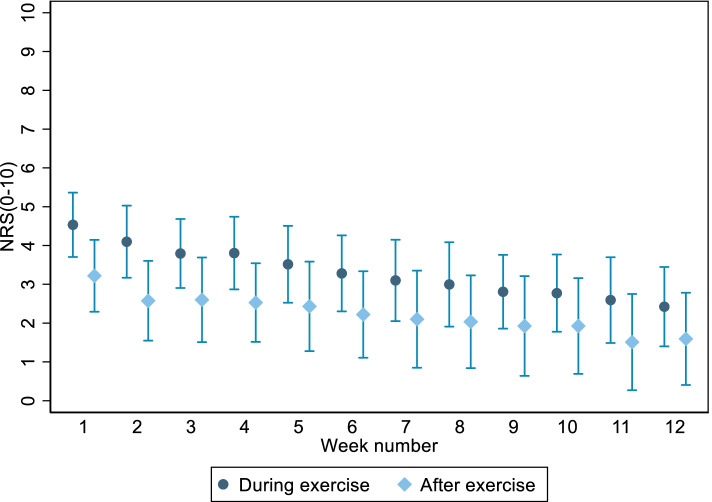
Mean pain scores during exercise and 2 h after completing exercise. Based on self-reported training-log data. NRS numeric rating scale

The pain response to the exercise intervention is displayed in Fig. 2 and indicated a gradual decrease in pain scores during the exercise intervention period. In 76% of all exercise sessions, patients reported NRS pain scores ≤ 5. Eleven out of 18 patients (61%) reported a NRS pain score above five. Of the sessions with NRS over five, 75% occurred during the first 6 weeks of exercise.

All patients progressed on level or load in at least one of the exercises during the 12 weeks of exercise; 44% of patients progressed in all five exercises during the 12-week intervention. A total of 83% of patients progressed in the external rotation exercises, 78% progressed in the internal rotation exercises, 89% progressed in the shoulder extension exercises, 78% progressed in the abduction exercises and 72% progressed in the flexion exercises.

Baseline, follow-up and change scores for each secondary outcome measures are reported in Table 4. Patients’ improvements measured by WOOS, DASH, SSV, VAS pain scores, and active ROM were statistically significant and clinically relevant [35–39].

**Table 4:** Patient-reported outcomes, shoulder range of motion, and use of analgesics before and after the progressive shoulder exercise intervention on 18 patients

Outcome measures	Baseline score Mean (95% CI)	Follow-up score Mean (95% CI)	Change score Mean (95% CI)
WOOS (0–100, 100 best)	42 (33; 52)	65 (55; 76)	23 (13; 33)^*^
DASH (0–100, 100 worst)	42 (35; 50)	30 (22; 37)	− 13 (− 19; − 6)^*^
EQ-5D-5L			
*Utility index (− 1, 1 best)*	0.66 (0.61; 0.72)	0.73 (0.67; 0.79)	0.06 (0.001; 0.14)
*VAS (0*–*100, 100 best)*	65 (54; 75)	73 (62; 84)	8 (6; 22)
Subjective shoulder value (0–100, 100 best)	45 (37; 53)	62 (53;71)	17 (5; 30)^**^
Pain (0–100, 100 worst)			
*VAS at rest*	51 (38; 64)	28 (17; 40)	− 23 (− 36; − 9)^**^
*VAS during activity*	66 (54; 77)	39 (25; 52)	− 27 (− 39: − 15)^*^
*Sleep disturbing pain (yes/no)*	17/1	9/8^a^	0.005^b^
AROM			
*Flexion, degrees*	110 (96; 125)	127 (112; 143)	17 (3; 31)
*Abduction, degrees*	99 (85; 112)	124 (110; 138)	25 (13; 37)
Usage of analgesics			
*Yes/no*	18/0	15/3	
*Daily/weekly/never*	10/7/0^a^	9/6/3	
*Paracetamol (yes/no)*	18/0	13/5	
*NSAIDs (yes/no)*	3/15	3/15	
*Opiods (yes/no)*	2/16	0/18	

*WOOS* Western Ontario Osteoarthritis of the Shoulder score, *DASH* Disabilities of the Arm, Shoulder and Hand, *VAS* visual analogue scale, *AROM* active range of motion

^*^Significant *p* value < 0.001

^**^Significant *p* value < 0.01

^a^Missing data on 1 patient

^b^Chi^2^

### Patient feedback

The patients reported the exercises strengthened their muscles and improved their performance of daily activities. Patients were willing to travel up to approximately 45 minutes to participate in the exercise intervention. In terms of the outcome measures, the patients found it relevant to measure shoulder-related symptoms, including pain at rest, at night and during activities and their ability to perform activities of daily living. Most of the patients reported to be willing to participate in a future RCT comparing shoulder replacement surgery with an exercise intervention.

## Discussion

The 12-week progressive shoulder exercise program for patients with glenohumeral OA and CTA eligible for total shoulder replacement was feasible, in terms of high adherence to the exercise program, acceptable reported pain levels in relation to the exercise program, no adverse events and a low number of dropouts. Further, clinically relevant improvements in patient-reported outcomes and ROM were found [35–38].

### Feasibility outcomes

The results regarding feasibility are similar to those in other feasibility studies. Our study found an adherence of 88% for supervised and 78% for home-based weekly exercise sessions, which is in accordance with adherence reported in other studies [69.9–91%] investigating exercise interventions in older adults with knee and/or hip OA [40–42]. In our study, patients reported NRS scores ≤ 5 in 76% of all exercise sessions. The patients experienced a gradual decrease in pain scores during the exercise period. This was similar to a study by Sandal et al. 2016 investigating the pain trajectory during eight weeks of neuromuscular exercise in patients with hip and knee pain [43]. Corresponding to a study on self-managed exercises in patients with persistent subacromial pain [44], no adverse events were registered in our study. Another feasibility study investigating heavy shoulder strengthening exercise in patients with shoulder hypermobility found 33.3% minor adverse events, consisting of short-lasting soreness, ache, “stuck” shoulder, headache and general soreness [25]. The lack of minor adverse events could be due to the underlying disease and different interventions between the studies. The dropout rate of 10% in this study is similar to other studies investigating feasibility of an exercise intervention on hip and knee OA where dropout rates varied from 1 to 33% [40–42]. The reasons for discontinuing the intervention were also comparable to other studies, i.e., increased pain or not related to the intervention.

Patients improved statistically significantly and clinically relevant on the patient-reported outcomes and on their active ROM [35–39]. However, it is important to interpret these findings with caution, as it is not possible to distinguish between effects that might have occurred due to the natural cause of the condition, regression to the mean, placebo, or the exercise intervention [45]. With regard to our feasibility measures and patient reported outcomes, the exercise intervention investigated in our study was feasible, safe, and well-balanced as patients completed the exercise intervention and improved on patient-reported outcome measures.

Although total shoulder replacement is an established treatment with a high patient satisfaction ranging from 75 to 100% [46], there is a need to further improve the treatment of patients with glenohumeral OA or CTA. In Denmark, there is a nationwide use of an evidence-based exercise program, called Good Life with osteoarthritis in Denmark (GLA:D), for patients with hip and knee OA [17]. A similar evidence-based standardized exercise program does not exist in the management of glenohumeral OA and CTA although several reviews on the management of glenohumeral OA and CTA suggest physiotherapy or exercise as the first line treatment [2, 15, 16, 47].

## Limitations

Due to the feasibility design, some limitations must be kept in mind when interpreting the results of the present study. It is a small study with methodological limitations, such as lack of a control group, thus the study has low statistical power, and no causal relationships were investigated. Of the 20 included patients, 15 had glenohumeral OA and only 5 had CTA [48]. The strengths of the study were the standardized, transparent, and precisely described exercise program and the pre-defined progression and evaluation criteria.

The current study highlights the feasibility and safety required before initiating a RCT comparing total shoulder replacement to progressive shoulder exercises.

## Conclusion

Progressive shoulder exercises are feasible and safe for patients with glenohumeral OA and CTA eligible for total shoulder replacement based on the recorded adherence, number of adverse events and pain response. The standardized exercise program was well balanced and seems to consist of appropriate exercises with the possibility to both regress and progress. The exercise program had a positive effect on shoulder pain, function, and ROM. A future RCT, will investigate the effectiveness of progressive shoulder exercises compared to shoulder replacement.

## Data Availability

The datasets generated and/or analyzed during the current study is available in anonymized form from the corresponding author on reasonable request.

## Abbreviations

OA: Osteoarthritis
CTA: Rotator cuff tear arthropathy
TSA: Total anatomical shoulder arthroplasty
RSA: Reverse shoulder arthroplasty
ROM: Range of motion
VAS: Visual analogue scale
NRS: Numeric rating scale
RCT: Randomized controlled trial
WOOS: Western Ontario Osteoarthritis of the Shoulder score
DASH: Disabilities of the Arm, Shoulder and Hand
SSV: Subjective shoulder value
